# Fistula

**Published:** 1880-06

**Authors:** G. Halsted Boyland

**Affiliations:** Baltimore, M. D.


ARTICLE IV.

### FISTULA.

BY G. HALSTED BOYLAND, M. A., M. D., ETC., BALTIMORE, MD.

Fistula in its widest signification, may be designated as a narrow, very deep seated ulcer, which from a deep, lying tissue, organ or cavity forms an abnormal communication with the surface of the outer skin or mucous membrane.

Fistula in its more strict sense is such abnormal canal or opening as leads to any normal or diseased secretory organs or their ducts, by means of which therefore, a portion of their secretions is continually emptied without or into another cavity. In distinction from this, other fistulas through which no special secretion but only pus flows may be classed as fistulous or canaliculated ulcers; these last being also imperfectly styled "blind sacks"—fistulae incompleted. If the opening of such a fistulous ulcer, is in the outer skin it is usually accepted as imperfect outer fistula; if on the contrary, the opening happens to be in a mucous membrane, as an imperfect inner fistula; this is probably overlooked in general practice, it being the custom of some to consider a fistula a fistula, and that is the end of it. But this distinction once marked, makes our therapeusis the more precise and effectual.

Perfect fistulae then have two openings, between which in different sizes the canal runs. This canal is so short sometimes as really not to be a canal at all; but only an abnormal opening; then, in other words, we have simply a hole that leads from one organ or cavity to another, or to the outside, e. g. tear sack fistulae; vesico-vaginal fistulae—the *lip formed* fistula of Roser. This name was probably chosen by Roser,* because in such cases the mucous membrane of one organ or cavity, is grown on to that of the other or the outer skin, just in the same manner as on the lips the mucous membrane grow to the skin of the face. But if there is a distinct fistulous passage, we have then to treat a regular canaliculated fistula; and such an one as we are most likely to meet with ordinarily. In this can be distinguished with certainty an inner and an outer opening. The inner opening of a canaliculated fistula is generally to be found in the middle of a somewhat indurated, slightly protruding ring, oftenest in a mucous membrane. Sometimes it is found on the summit of a small prominence or buried in a deep fold of the mucous membrane, seldomer between scar strings. Rarely instead of one opening are several present.

The outer opening is often only uncommonly small and narrow, and therefore difficult to find. At times it is surrounded by soft spongy growths, which bleed at the slightest touch. It can also be situated upon a little red knob which is at times conical, at others with a pedicle like a polypus. In other cases, the outer opening is to be found in the bottom of a funnel. This phenomenon is produced by the membrane of the fistula, which draws itself down partaking of the nature of cicatricial tissue, to gradually shorten; or by swelling of the surrounding parts, whilst the fistulous passage does not lengthen. The outer opening is often double or triple. The course of the fistula is generally not on a straight line, often very tortuous; some even run zig-zag. When several outer openings are present, the fistula corresponding to these, is branched. Not unfrequently in the course of a fistulae are found great cavities or sinuses, especially when the fistula was already old or was formed by several abscesses having run into each other. These are then of especial importance when they communicate with a diseased bone. The *communication fistula* technically speaking, however, is one that does not open on the outer skin, but leads from one cavity covered with mucous membrane to another such. In these it can, of course, only be a question of a relative outer and a relative inner opening according to the more easy accessibility

*Roser, Chirurgie.

from without. But these are generally lip-formed fistulae so that both openings form together one. The course of the fistula is covered with a membrane analogous to the abscess membrane, and which at first sight appears very much like a mucous membrane. The same is of a bright red color on account of its richness in capillary vessels, secretes a pus more or less like mucous, but has no villi nor characteristic epithelium. Sometimes it is surrounded by a layer of thickened connective tissue, analogous to submucous tissue; but ordinarily it hangs very closely together with the surrounding parts. It may develop itself everywhere, in the most different tissues. If it goes into inflammation the secretion either stops entirely or is at least altered; whilst it is otherwise almost devoid of sensibility, it then becomes exceedingly sensitive. It possesses great tendency to shorten, in like manner to, cicatricial tissue. On the other hand, its opposite surfaces have as little tendency as mucous membranes to grow together. In the immediate surrounding of fistulous passages is generally found a considerable hardness, callosity, a true induration as sequence to chronic inflammation, which either has taken place around the fistula or is still going on. The connecting tissue especially, has lost its elasticity, appears on section of whitish opal color, sometimes like fat, and is easily cut into layers. Not unfrequently we find that the fistulous canals can conduct liquids only in one direction; at one time, from within, outward, so that no air can press in, (this can be useful,) at others, the reverse, so that the emptying of pus and other liquids flowing in the fistula, thus encounters hinderance. Roser called attention to the presence of a valve like formation as an explanation of this condition; sometimes the protuberance of isolated granulations or folds form a kind of valve, sometimes the oblique direction in which the fistulous canal pierces the skin has this effect, finally the closing may be caused by the fistulous opening being on the top of a heap a granulations, whose compression by a liquid pressing against it, compromises at the same time the course of the fistula itself.*

*Ætiologie.*—The formation of a perfect fistula from congenital fistula (malformation), always pre-supposes the opening of a cavity covered with mucous, secreting membrane or containing a secretion. This can take place either from without or from within. The formation of a fistula may then be reduced: 1st, to wounding of a secreting

*Roser, Ueber abscess and fistelklappen. Archir for physiologische Heilkunde. p. 349.

organ or an excretory duct from without, the healing of which, on account of adhesion, takes place only partially or not at all. 2d, Breaking through inwardly of an abscess that has formed near a gland, a duct or any cavity in general, covered with mucous membrane—such abscess forcing itself through the walls of the parts in question and inwardly, because situated under fast fibrous membranes that either prevent entirely or at least hinder it from breaking outwardly; 3d, Tearing of a duct and infiltration of its contents into the surrounding tissue. 4th. Ulceration on the mucous membrane of a given organ, which little by little eats through its walls, and finally, in the same manner as tearing, brings about an outflow of the contents and an attendant ulceration that continues until the outer skin is broken through. The last method of formation is favored by every hindrance that may oppose the emptying of secretions in the natural way. Narrowing of a duct favors especially the formation of a fistula on the way between the secreting organ and the narrowed place.

If either of these manners of formations takes place, these are all the same with reference to a completion of the fistula, three possibilities: 1st. A perfect fistula is formed at once, as for example, by a wound, or the breaking of an abscess outwardly and inwardly at the same moment. 2d. An imperfect inner fistula is first formed, for instance, by ulceration going out from the mucous membrane or by tearing of a duct. 3d. An imperfect outer fistula first formed, e. g. by a wound piercing to the neighborhood of a secreting cavity, or by an abscess that is formed near it, but first broken outward. It is understood very easily how in the two last mentioned cases by the further advancing ulceration, an imperfect fistula can nearly be converted into a perfect one. In the formation of many fistulae the influence of a corroding secretion plays an important part in that of others, the existence of a dyscrasia. Ulcerations of mucous membranes generally rest upon a dyscrasia. Wounds without loss of substance and without crushing of the adjacent parts, are followed by the formation of fistulae, only under the influence of a corroding secretion or of a dyscrasia.

For the above technical classification the profession is indebted to Prof. Bardeleben of the Berlin University, whose skill as teacher and operator, it was my good fortune to witness by ear and eye, during a course years ago, at the Berlin University. This classification has a more than technical value on account of being so very practical. A cause of fistula often overlooked, is hemorrhoids; when bleeding piles are neglected or when there exist both external and internal piles, together these being neglected or badly treated, sometimes result in fistula—brought about by the breaking down of peri-anal tissue. As regards the class of fistulae usually met with by the general practitioner, the fistula in ano seems to be the most frequent. Vesico-vaginal and recto-vaginal fistulae are probably those that he would meet with the least frequently, indeed, he might live to great age and not have to deal with them at all.

*Prognosis.*—The more important the organ with which the fistula communicates, the more necessary for life, the fluid that flows from it, the greater the danger. The chances of healing are the better, the smaller and fresher the fistula is, the easier we can procure for the secretions a perfect issue in the normal way, finally the less the general condition and nutrition of the patient are disturbed, either by a dyscrasia or otherwise. A lip-formed fistula never heals without surgical interference. A canal-formed fistula only heals when its ætiological moments have ceased, and a sufficiently strong and progres sive cicatricial contraction take place in it.

*Treatment.*—The indication is very suggestive. It would be enough for the ordinary mind to see, any abnormal opening to comprehend that, as a general rule, such opening ought to be closed. But to close a fistula the ordinary means of procuring re-union are not sufficient; it is rather required, on the one hand, to make free again the normal passage of the secretion flowing through the fistula; to widen the natural passage (duct) or even sometimes to make a new one at the place of the closed or narrowed one; sometimes to close a part of it, at others, to make a clean, fast union of the walls of the fistula. Between closing an open wound and converting a, so to speak, closed wound into an open one, there is room for a great variety of procedures, from which the physician will select the one most appropriate to the case he may be called upon to treat; there are, therefore, mostly operative measures at times of greater, at others of less importance, necessary in the treatment of fistula. Notably in lip-formed fistula the lips as a rule must be cut off in order to produce fresh bleeding wound borders that must be secured by sutures; fistula-canals must be cauterized (when it is deemed best to employ cautery) repeatedly and not only within the fistula, per se, but in a free manner going slightly into the surrounding tissue, in order to induce by exciting concentric cicatricial contraction, a gradual closing. If the fistulous passage is situated on the surface, it is best to split it with a bistoury or galvano-cautery, and then to let it heal by granulation. When these simple operations can be carried out in the reasonable anticipation of a good result, and there is no danger to be feared as to the life of the patient, it would be useless indeed, to either *seriously* employ or even first try in its stead a pharmaceutical course, which only perhaps, after a long employment and by a great detour, might reach the same end. Shall we operate then all fistulae? Is it conformable to our purpose in general, to heal all fistulae?

If the fistula causes considerable loss of a secretion necessary to life, it must invariably be operated. Here belong the majority of intestinal fecal fistulae. Other fistulae cause so much misery to the patient's self and surroundings, and make his life so unbearable, that in this case also a radical operation is rightly desired on the part of the patient and rightly recommended on the part of the physician. Here belong the majority of urinary fistulae, especially the vesico-vaginal fistula. Also salivary and lachrymal fistulae can make the patient so irritable and miserable that he himself wishes them operated upon; a wish that is to be the more readily gratified as both operations are usually without danger. On the other hand, those fistulae which serve to conduct off diseased matter (like a fontanel,) ought not, according to the elder authorities with whom some later ones also agree, to be operated upon; when, for example, an old man has a fistula in ano, and at the same time is affected with any inner disease, complaining of a feeling of heaviness in the head, cough, shortness of breath, torpid digestion; we should consider it dangerous to life to operate on account of the arrest of habitual secretions. We would be the more willing to support their view when the other symptoms improve as the fistula develops itself, a condition that can readily occur. If, however, the other troubles remain in spite of the fistula, there is no reason for not removing it, provided the operation would not be attended with marked danger in any individual case; for instance, in an old individual suffering from diseases, which by long lying still, would be made worse, and obliged to keep his bed for as long a time as would be necessary after an operation, whilst he could go about with the fistula, we must certainly refuse an operation; not so much on account of the dangers that might arise from the healing of the fistula as on account of the lying still. Again, in consumptive patients, if there is a probability of the operation for fistula having fever as a consequence, which in the course of the disease, would be grave, we should just as certainly refuse the operation here as in every case in which it would induce fever. As regards vesico-vaginal and recto-vaginal fistula, these are cases in which the general practitioner might entertain doubts as to undertaking them, if he had not the assurance which is here given that the operation only requires patience, care and a good set of instruments made for the purpose, that can be obtained from any instrument maker. Sims' instruments are the best; of course his speculum is the only one that will do under the circumstances. Two or three assistants are better. The patient being placed in the most convenient position (without regard to rule,) for the operation, the borders of the fistula are freshened up and then united, the patient being kept under the influence of chloroform. If undertaken under favorable conditions, the operation is usually successful; the after treatment requiring much care; the bladder should be emptied regularly and attention given to the diet. Hewitt advises leaving a catheter in the bladder after the operation for vesico-vaginal fistula. After the operation for recto-vaginal fistula the principal point is to keep the bowels confined for some days, by means of opium, until cicatrication is well under way. Freshening up the borders of any fistula is not always followed by a cure as witness the following case from my own practice: L. S. aged 19, of nervo-sanguineous temperament had had a swelling of the glands of the cutis, on the cheek; these had formed a sort of knot which had been extirpated before I saw him. After the operation the wound (being one about an inch in length,) was stitched up, but whilst the process of healing was going on, he accidentally knocked out the stitch nearest the nose, and the wound healed, leaving an ugly three cornered fistulous hole in his cheek, through which the deep layer of cellular tissue could be seen a yellowish mass of the color of bone, which I at first thought it was. When he came to me the borders were very tough and hard, I freshened up the borders with the bistoury to get a good healing surface for primam, and laid on one suture, which was taken out on the third day, when the wound seemed to be doing well. He went away and was not seen by me again, but I afterwards learned that the skin parted and the fistulous opening returned; so tough and fibrous had been the borders. This patient suffered no inconvenience other than bad appearance, as there was no discharge and the cellular tissue formed a good elastic and dry bottom.

From the foregoing notes, it will be seen that the general ground of fistula is covered, and that the principle, although broad and fixed, must nevertheless, be applied in manner such as each individual case may suggest itself to the practitioner, who will be best guided not so much by any fixed rules, as by the exigencies that may present themselves; varying often both in nature and position, as well as in form. The strict observance of this will be followed by the most speedy good results and ensure at the same time the most scientific and practical treatment of fistula.

